# Virtual Excitation and Multiple Scattering Correction Terms to the Neutron Index of Refraction for Hydrogen

**DOI:** 10.6028/jres.110.035

**Published:** 2005-06-01

**Authors:** K. Schoen, W. M. Snow, H. Kaiser, S. A. Werner

**Affiliations:** University of Missouri-Columbia, Columbia, MO 65211, USA; Indiana University/IUCF, Bloomington, IN 47408, USA; University of Missouri-Columbia, Columbia, MO 65211, USA

**Keywords:** neutron interferometry, neutron optics, Nowak correction, refractive index, scattering length

## Abstract

The neutron index of refraction is generally derived theoretically in the Fermi approximation. However, the Fermi approximation neglects the effects of the binding of the nuclei of a material as well as multiple scattering. Calculations by Nowak introduced correction terms to the neutron index of refraction that are quadratic in the scattering length and of order 10^−3^ fm for hydrogen and deuterium. These correction terms produce a small shift in the final value for the coherent scattering length of H_2_ in a recent neutron interferometry experiment.

## 1. Neutron Optics Theory

The basis of neutron optics is the Schrödinger equation for the coherent interaction of the neutron with matter using an optical potential. It is well known that the wavefunction satisfies the Lippmann-Schwinger scattering equation [1]

Ψ(r)=|k〉+Gv(r)Ψ(r) (1)

where *ψ*(**r**) is the wavefunction, |*k*〉 is the incident wave, *G* is the one-body Green’s function, and *v* is the optical potential. It is related to the *t*-matrix by

t=ν(r)+tGν(r). (2)

Thus, given a form for the *t*-matrix, one can determine the optical potential and use this to solve the Schrödinger equation for the form of the wavefunction.

The form for the *t*-matrix used in the Fermi approximation, the standard approach to solving for the optical potential in the theory of neutron optics, is

$$t=\sum_{j}t_{j},$$ (3)

where

$$t_{j}=\frac{2\pi \hbar^{2}}{m}\sum_{j}b_{j}\delta \left(r-R_{j}\right).$$ (4)

In the above equation, *j* denotes the atomic species, *m* is the neutron mass, *b_j_* is the coherent scattering length, *δ* is the delta function, **r** is the coordinate of the neutron, and **R***_j_* is the coordinate of the atomic scattering center. Using this in conjunction with the Born approximation, *t* = *v*, the optical potential then becomes

$$\nu \left(r\right)=\frac{2\pi \hbar^{2}}{m}\sum_{j}N_{j}b_{j},$$ (5)

where *N_j_* is the atomic density.

This leads to the familiar form of the index of refraction for the Fermi approximation,

$$n^{2}=1-\frac{4\pi }{k^{2}}\sum_{j}N_{j}b_{j}.$$ (6)

However, this approximation disregards atomic binding and multiple scattering. Thus Eq. (3) must be modified to take these effects into account. The next order term in the multiple scattering expansion of the *t*-matrix is

$$t=\sum_{j}t_{j}+\sum_{j,j\prime j\neq j\prime }t_{j}Gt_{j\prime }.$$ (7)

In general this correction term to the *t*-matrix is difficult to evaluate. However, in the particular case of molecular hydrogen and deuterium gas, one can perform this calculation in the rigid rotor approximation. The two correction terms for H_2_ gas are

$$\nu_{bp}^{\left(2\right)}\left(k\right)=-\frac{iN}{{\left(2\pi \right)}^{3}\hbar }{\left(\frac{2\pi \hbar^{2}}{m}\right)}^{2}\int_{-\infty }^{+\infty }dtt_{+\eta }^{-\frac{3}{2}}w_{-0}^{\frac{3}{2}}\left(t\right)e^{-\frac{\varepsilon }{\hbar }\left|t\right|}\int d^{3}qe^{i\frac{\hbar t}{2m}\left(k^{2}-q^{2}\right)}\frac{1}{N}\times \sum_{j}\langle b_{j}^{2}e^{i\left(q-k\right)R_{j}\left(t\right)}e^{i\left(k-q\right)R_{j}\left(0\right)}\rangle ,$$ (8)

and

$$\nu_{ms}^{\left(2\right)}\left(k\right)=-\frac{iN}{{\left(2\pi \right)}^{3}\hbar }\left(\frac{2\pi \hbar^{2}}{m}\right){\int_{-\infty }^{+\infty }dte^{-\frac{\varepsilon }{\hbar }\left|t\right|}\int d^{3}qe}^{i\frac{\hbar t}{2m}\left(k^{2}-q^{2}\right)}\frac{1}{N}\times \sum_{j,j\prime j\neq j\prime }[\langle b_{j}b_{j\prime }e^{i\left(q-k\right)R_{j}\left(t\right)}e^{i\left(k-q\right)R_{j\prime }\left(0\right)}\rangle -\langle b_{j}e^{i\left(q-k\right)R_{j}\left(t\right)}\rangle \langle b_{j\prime }e^{i\left(k-q\right)R_{j\prime }\left(0\right)}\rangle ],$$ (9)

due to the binding potential and multiple scattering respectively. These potential terms may be expressed in terms of the index of refraction,

1−Ren2=4πNb¯k2+mℏ2k2(Revbp(2)(0)+Revms(2)(0))≡4πNb¯k2(1+Δbp+Δms). (10)

If one assumes a rigid rotator approximation for the rotational excitations of the hydrogen molecule, the potential terms can be numerically integrated. Nowak performed these calculations [3], and his results are seen in Table 1.

## 2. Experimental Procedure

**Fig. 1:**
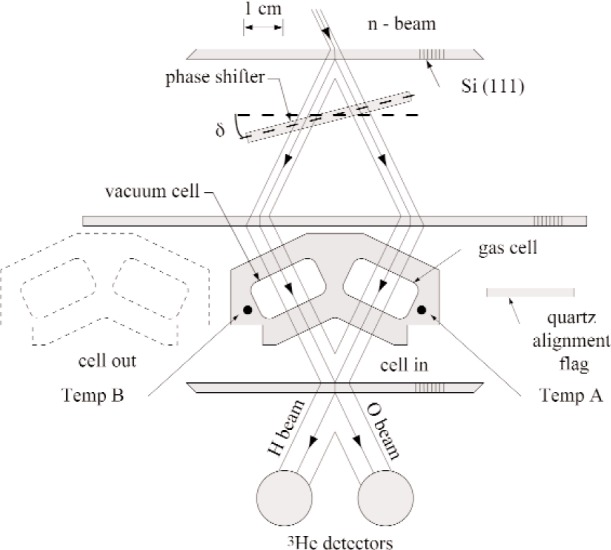
A schematic view of the Si perfect crystal neutron interferometer with gas cell.

The measurement of the real part of the n-p coherent scattering length was performed at the National Institute of Standards and Technology (NIST) Center for Neutron Research (NCNR) Interferometer and Optics Facility using a triple-blade, single perfect crystal silicon neutron interferometer. See Fig. 1. A more detailed description of the method, experimental arrangement and procedure for the precise determination of coherent scattering lengths using neutron interferometry can be found in Schoen et al. [2].

The phase shift that occurs between the two coherent neutron beams due to the presence of the H_2_ gas along one of the paths is given by

$$\Delta \phi =\left(n-1\right)kd=-Nb_{np}\lambda d,$$ (11)

where Δ*ϕ* is the phase shift, *n* is the real part of the index of refraction, *k* is the wavevector, *N* is the atomic density, *λ* is the neutron wavelength, and *d* is the path length that the neutron beam traverses through the H_2_ gas. From this relationship the coherent scattering length can be determined.

A secondary sampling method is used to measure the phase shift. This is accomplished by positioning a quartz phase shifter across the two beams traversing the interferometer and rotating the angle, *δ*, about the mounting axis. The intensities of the beams that arrive at the ^3^He detectors, labeled O beam and H beam as in the diagram, are a function of the phase shifter angle, and are given by

$$I_{O}\left(\delta \right)=A_{O}+B\;\cos\left(Cf\left(\delta \right)+\phi_{0}\right)$$ (12)

$$I_{H}\left(\delta \right)=A_{H}+B\;\cos\left(Cf\left(\delta \right)+\phi_{0}+\pi \right).$$ (13)

The function *f*(*δ*) is given by the path length difference between the two beams travelling through the phase shifter. *A*_O_, *A*_H_, *B*, *C*, and *ϕ*_0_ are parameters that are used to fit the data.

**Fig. 2:**
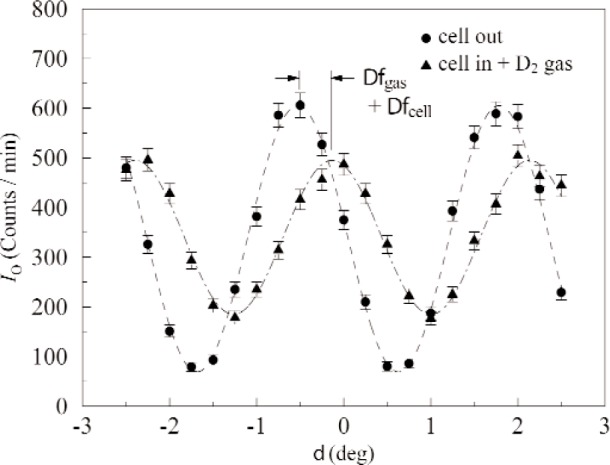
An example of an interferogram in which the intensity changes as the phase angle *δ* is varied. Data is shown with both the gas cell in the interferometer and outside.

A gas cell with two chambers, specifically designed to minimize the phase shift due to the presence of the cell itself by spanning both beams, held one chamber filled with 99.999 % chemically pure H_2_ gas from Matheson Tri-Gas, and the other chamber was evacuated. This cell was placed in the interferometer, after its mounting had been aligned using a quartz alignment flag, such that one beam encountered a vacuum along its path length, while the other encountered the H_2_ gas. The phase shift caused by the gas was determined by collecting interferograms. See Fig. 2. First an interferogram was measured with the gas cell in the interferometer, and the angle of the quartz phase shifter was varied. The phase was determined from the data fitting. This was then repeated with the gas cell out of the interferometer, to obtain the interferometer offset phase. The difference between the two phases is due to the presence of the gas and the cell. The phase shift due to the cell itself was determined by holding both chambers evacuated, and this value was subtracted from the gas and cell measurement to yield the phase shift due to the gas alone. This procedure of measuring the phase shift due to the gas was repeated approximately 350 times.

The atomic density was determined using the virial equation up to the third virial coefficient. The second and third virial coefficients were obtained from the compilation by Dymond [4]. In addition to the correct coefficients, the virial equation also requires accurate knowledge of the temperature and pressure. The temperature was monitored and recorded using 100 Ω platinum resistance thermometers, and the pressure was measured using a silicon pressure transducer as in previous scattering length measurements [2].

The thickness of the gas cell was measured to be (1.006 ± 0.0001) cm by the NIST Precision Engineering Division Coordinate Measuring Machine [5]. This thickness was corrected for thermal expansion and contraction due to temperature fluctuation (*α* = 2.5 × 10^−5^ °C^−1^ for aluminum) for each individual run. The effect of the gas pressure (≈12 bar) on the thickness of the cell was negligible.

The wavelength of the neutrons traversing the interferometer was measured using a pyrolytic graphite (PG 002) crystal. This analyzer crystal was placed in the H-beam of the interferometer and rotated about until both the symmetric and anti-symmetric Bragg reflections were found. Bragg’s law, *λ* = 2*d* sin*Θ*_B_, was then used to determine the actual wavelength. For this experiment, *λ* = (0.2713050 ± 0.0000085) nm, and, in a separate test, the stability of the wavelength over time was shown to be 0.001 %.

**Fig. 3:**
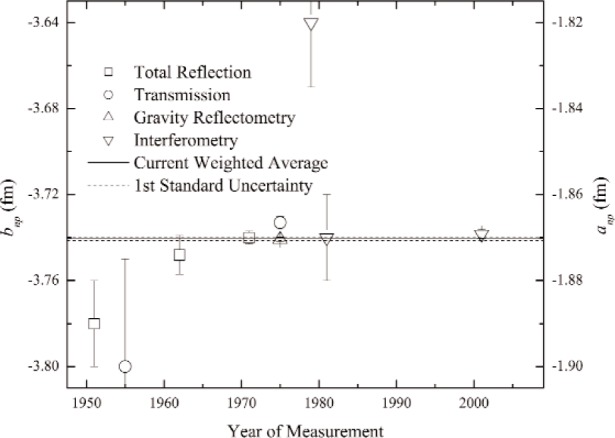
A plot of the bound coherent neutron scattering lengths for the n-p system along with reported uncertainties. Our result for H_2_, neglecting the Nowak correction term, is 2.3 *σ* away from the world average of experimental results. Inclusion of the theoretical correction brings our value into closer agreement, as can be seen.

The value of the coherent scattering length was calculated for each data set on a run-by-run basis, and then averaged to obtain a final value of *b*_H_ = (−3.7458 ± 0.0020) fm. This result uncorrected, for the correction terms discussed above, differed from the world average of previous results, *b*_H_ = (−3.7410 ± 0.0010) fm, by approximately 2.3 *σ*. The highest-precision previous measurements of the coherent n-p scattering length, conducted using a gravity refractometer using neutron reflection from liquid hydrocarbons, require a negligible correction for higher order contribution to the *t*-matrix due to the larger mass of the molecules. From Eq. (10), it is observed that the Nowak correction term simply adds to the nuclear scattering length. Therefore, in determining the n-p scattering length, the observed scattering length for the neutron-hydrogen system was simply divided by (1 + *Δ_bp_* + *Δ_ms_*). Our result became *b_np_* = (−3.7384 ± 0.002) fm, assuming no error in the theoretical calculation. This agrees with the previous experimental values (see Fig. 3). A higher precision measurement of the n-p scattering length using H_2_ gas at different temperatures and ortho-para ratios could be used to experimentally isolate these correction terms.

## Figures and Tables

**Table 1:** Average corrections to the refractive index for H_2_ gas

Temperature (K)	$\bar{\Delta }=\Delta_{ms}+\Delta_{bp}$
100	2.0 × 10^−3^
300	1.9 × 10^−3^
